# Survivorship preparedness and activation among survivors of lymphoma

**DOI:** 10.1007/s11764-024-01664-6

**Published:** 2024-08-27

**Authors:** Sharon L. Manne, Shawna V. Hudson, Dena O’Malley, Katie A. Devine, Matthew Matasar, Jacintha Peram, Justin Solleder, Elizabeth Handorf, Andrew M. Evens

**Affiliations:** 1 Rutgers Cancer Institute of New Jersey, 120 Albany Street, Tower 2 Floor 8, New Brunswick, NJ 08901 USA; 2 Dept Family Medicine and Community Health, Rutgers RWJ Medical School, 303 George Street, Rm 309, New Brunswick, NJ 08901 USA; 3 Rutgers Cancer Institute of New Jersey, 195 Little Albany Street, New Brunswick, NJ 08901 USA; 4 Rutgers School of Public Health, 120 Albany Street, Tower 2 Floor 5, New Brunswick, NJ 08901 USA

**Keywords:** Cancer, Lymphoma survivorship, Oncology, Patient activation, Survivorship preparedness

## Abstract

**Objectives:**

Taking an active role in managing post-treatment care has emerged as a key aspect of promoting a successful transition into survivorship and is associated with better patient outcomes. In this study, we focus on two key aspects of active self-management, activation and preparedness. Activation was defined as understanding one’s role in the care process and having the knowledge, skill, and confidence to take on a role in managing self-care. Preparedness was defined as the extent to which individuals perceived they had sufficient information about what to expect after cancer treatments are completed. The study goal was to characterize survivorship preparedness and activation among lymphoma survivors within 5 years of treatment completion in New Jersey and examine the association of sociodemographic, medical, care transition experiences, practical concerns, and psychosocial factors with activation and preparedness.

**Methods:**

One hundred and one Hodgkin lymphoma or non-Hodgkin lymphoma survivors who had completed treatment within 5 years completed a survey of survivorship care experiences (response rate = 34.12%).

**Results:**

Approximately 60% of survivors reported high activation, with similar percentages for higher preparedness. Less activated survivors were significantly (*p* < .05) younger, married, resided in a more deprived geographic area, and reported more fatigue and information needs. Less activated survivors reported recalling that their providers were significantly (*p* < .05) less likely to discuss long-term side effects, psychosocial needs, risk-reducing lifestyle recommendations, and how to manage other medical concerns. Fewer care transition practices were most strongly associated with lower preparedness.

**Conclusions:**

A significant proportion were not activated for survivorship, and both activation and preparedness were strongly associated with providers’ survivorship transition practices.

**Implications for Cancer Survivors:**

Implementing programs to foster more activation and preparedness for lymphoma survivorship care would benefit from education about recommended follow-up care and healthy lifestyle practices. Providers should routinely ask about their patients’ confidence and preparedness for survivorship and provide referrals for appropriate care as needed.

The transition from acute care into survivorship is a critical point in the survivorship trajectory where many patients experience a loss of formal support for care [1]. During this transition, patients are expected to manage their care, including adhering to recommended surveillance and follow-up; managing physical, practical, social, and emotional effects; and adopting healthier habits. These tasks can be challenging as the healthcare system has become more fragmented. Since the duration of survivorship is lengthy for most, it is important to prepare them for potential challenges and health practices that may foster a better quality of life.

Being active in managing care is an important aspect of successful survivorship. Patient activation is broadly defined as understanding one’s role in the care process and having the knowledge, skill, and confidence, to take on a role in managing self-care [2–4]. In the non-cancer context, greater activation has been associated with important outcomes including medication adherence [5], higher quality of life, and physical and mental functional status [5, 6]. Among cancer survivors, activated patients are more proactive and engaged in managing their cancer [7], feel their treatment plan reflects their values and goals [7], report lower symptom distress [8], and report higher engagement in exercise and adoption of a healthier diet [7].

A second key aspect of successful survivorship is preparedness, which is typically defined as the extent to which an individual perceives she/he has sufficient information about what to expect after primary cancer treatments are complete [9]. The significance of preparedness is supported by research suggesting it plays a role in clinical outcomes such as higher perceived quality of care [10], symptom management self-efficacy [10], and quality of life [11]. Further, studies suggest survivors who report lower levels of preparedness may derive more benefits from interventions that address symptoms and concerns than those who report higher preparedness [12].

There is limited literature supporting the efficacy of survivorship transition interventions, with studies evaluating interventions for breast cancer survivors [13, 14], colorectal cancer survivors [15, 16], and head and neck cancer survivors [17]. These studies have included psychoeducation, training in managing specific adverse effects, self-management strategies, and developing skills to manage symptoms. Given their importance, characterizing activation and preparedness and identifying the sociodemographic, medical, care transition experiences, practical concerns, and psychosocial factors associated with preparedness and activation has the potential to foster a greater understanding of this construct and facilitate the development of effective interventions to improve these modifiable risk factors. There are no studies evaluating activation and preparedness or their correlates among lymphoma survivors and limited literature evaluating correlates of patient activation among survivors of other cancers. A study of breast and prostate cancer survivors reported that non-Caucasian race, being unmarried, unemployed, lower income, less time with clinicians, and higher fear of recurrence were associated with less activation [18]. Mazanec and colleagues [8] found that lower activation was associated with more fatigue and depression, greater work impairment, poor perceived health, and lower quality of life. Higher activation was associated with being single or living alone [8]. In terms of preparedness, lower preparedness has been associated with sociodemographic factors, such as not being married/partnered [19], female sex [10], financial hardship and residential census-level deprivation [20], medical factors [10], survivorship care transition practices [10], and psychological factors [10].

This study aimed to address the gap in understanding activation and preparedness among lymphoma survivors. Lymphoma is a cancer of the lymphatic system with two overarching subtypes, non-Hodgkin lymphoma (NHL) and Hodgkin lymphoma (HL). Although the long-term prognosis of many lymphomas is excellent, survivors can experience significant physical, practical, social, and emotional challenges. Common concerns include the loss of muscle strength, fatigue, sleep disturbance, and cognitive changes [21–27]. Potential late effects include an increased risk of heart, lung, and kidney disease, particularly among survivors with pre-existing comorbidities [28, 29]. These risks can contribute to health worries and compromise quality of life (QOL). We had two aims. The first aim was to characterize activation and preparedness using descriptive statistics. The second aim was to examine the association of sociodemographic, medical, and care transition experiences (e.g., patient recalled discussion about reasons for follow-up care); practical concerns (e.g., cancer-related financial hardships, information needs); and psychosocial factors (e.g., support needs, cancer concerns, anxiety, depression, fear of recurrence) with activation and preparedness. The ultimate goal was to identify lymphoma survivors at greatest risk of low activation and preparedness to inform future interventions.

## Methods

The study used a cross-sectional design with participants offered an online or paper survey.

### Eligibility

Prospective participants were eligible for this study if they (a) were between the ages of 18–80, (b) had been diagnosed with Hodgkin’s or non-Hodgkin’s lymphoma and completed any form of treatment within the last 5 years, (c) had completed active treatment and been informed that they are in remission, (d) had sufficient vision to read and complete a survey, and (e) could read English.

### Procedure and participants

This study was conducted at Rutgers Cancer Institute from the adult lymphoma service. The study received institution review board approval from the host institution prior to study commencement and conformed to recognized standards of United States Federal Policy for the Protection of Human Subjects. Prospective participants were identified using a search of the electronic health records of all patients seen by the Lymphoma Service. Prospective participants were mailed a recruitment package, which included a cover letter, study information, consent form, paper questionnaire, and postage-paid return envelope. If they wished, participants were offered both a paper consent and survey and an online consent and survey. Surveys were mailed out between October 2022 and November 2023. One week after the information package was mailed, individuals were called by a staff member to confirm receipt of the recruitment package, determine eligibility, and answer any questions about the study. Interested patients completed the online or paper consent form and questionnaire, but all participants opted for the online consent and survey. Prospective participants were called 1–2 times per week at varied days and times to increase the potential of reaching the patient. There was a maximum of eight calls. Potential participants who could not be contacted were considered passive refusers. When reached, individuals who did not agree to participate were considered active refusers. In addition to follow-up phone calls, text and email reminders were sent to prospective participants. Participants received a $50 gift card as an incentive. Surveys took between 30 and 45 min to complete.

Of the 350 individuals assessed for eligibility and study interest, 54 were ineligible, 195 refused, and 101 participants were recruited (34.12% response rate). Comparisons of the 101 acceptors and the 195 refusers based on available data (sex, ethnicity, age, age at diagnosis, time since remission, cancer type) indicated one difference. Participants with non-Hodgkins’ lymphoma (NHL) were more likely to refuse (70.6%) than those with Hodgkin’s lymphoma (55%) (chi-square = 6.311, *p* < 0.05). No other differences were seen.

### Measures

#### Sociodemographic domain

Self-reported sociodemographic factors included age (years), sex, ethnic background, racial background, marital status, education, and employment status. Participants indicated whether they were born in the USA. Participant zip code was used to assess area-level poverty level [30, 31]. Data were obtained from the US Census 2010.

#### Medical domain

Cancer variables collected from the medical record included lymphoma type (Hodgkin, non-Hodgkin’s), date of diagnosis, and treatments received. Time since diagnosis and time since remission were calculated and used in the analyses. Other medical variables included comorbidities, fatigue, and sleep quality. Participants completed a checklist of 23 comorbidities derived from the Health Information National Trends Survey (HINTS) [32, 33]. Frequency of *yes* responses was used to score the measure. Participants completed the Piper Fatigue survey-short form [34, 35], a 12-item scale with 11 response categories on a 0 to 10 metric. An average score was calculated. Coefficient alpha = 0.96. Sleep disturbance was evaluated using the Jenkins Sleep Scale [36]. Four items assess the frequency of each of the following problems during the previous 4 weeks: difficulties falling asleep, frequent awakenings during the night/able to fall back asleep, frequent awakenings/not able to fall back asleep, and non-restorative sleep. An average score was calculated. Higher scores indicate more sleep problems. Cronbach’s alpha = 0.68.

#### Survivorship transition practices domain

##### Care transition practices

Four items from the Follow-up Care Use and Health Outcomes Survey (FOCUS) [37] assessed whether participants recalled: (1) being informed that they needed regular follow-up care and monitoring following treatment completion; (2) receiving a written summary of the treatment; (3) receiving instructions from a doctor, nurse, or other health professional about follow-up appointments or who the participant should see for routine cancer checkups following treatment; and (4) being provided access to a patient navigator before or after their cancer diagnosis. Responses included *yes*, *no*, and *I don’t know*. The frequency of *yes* responses was used to score the measure.

##### Discussions about reasons for follow-up care

Eight reasons adapted from the FOCUS: [37] (e.g., “To check for a recurrence of the original cancer”). The number of responses checked yes was summed for the score, with higher scores indicating more reasons discussed.

##### Discussions about late effects, social and emotional needs, and lifestyle recommendations

Four items from the HINTS [32, 33] assessed the degree to which a care provider discussed: (1) managing other medical concerns; (2) late or long-term side effects of cancer treatment the participant might experience over time; (3) emotional or social needs related to the participant’s cancer, its treatment, or lasting effects of treatment; and (4) lifestyle or health recommendations such as diet, weight control, exercise, and quitting smoking. Items were rated on a 3-point scale. The average was calculated. Higher scores indicated higher levels of discussion. Cronbach’s alpha = 0.80.

#### Practical domain

##### Cancer-related financial hardship

Five items assessed financial hardships experienced due to the participant’s cancer diagnosis, treatment, and/or lasting effects, and included the following: (1) had to borrow money or go into debt, (2) had to make financial sacrifices, (3) had to declare bankruptcy, (4) worry about paying large medical bills, and (5) unable to cover their share of the medical care visits. All items were rated on a dichotomous scale. The number of *yes* responses was counted with a higher score indicating hardship [38].

##### Information needs

A 12-item scale was adapted from the Information about Health- Related Topics section of the FOCUS [37] and a review of the literature on the side effects of cancer. Topics included cancer-related follow-up tests the patient should have and cancer symptoms that should prompt calling a doctor. Responses were rated *yes* or *no*, and frequencies were calculated for the total score. Cronbach’s alpha = 0.88.

#### Psychosocial domain

##### Support needs

The Supportive Care Needs Survey [39] is a 34-item scale that assesses physical, psychological, and healthcare system needs. Items are rated on a 5-point Likert scale. An average was calculated as the number of needs rated as a “*moderate need*” or “*high need*.”

##### Fear of recurrence

The Concerns about Recurrence Scale (CARS) [40] is a 4-item scale assessing worries about the possibility of cancer recurrence. Items are rated on a 6-point Likert scale. Items were scored using mean across all items with higher scores indicating greater fear. The measure has been validated in other cancer survivor samples for internal consistency [40]. Cronbach’s alpha = 0.93.

##### Cancer concerns

An 11-item scale adapted from our prior work [41] assessed cancer-related concerns (e.g., physical appearance, emotional reactions, job-related concerns). Participants rated the degree of concern on a 6-point Likert scale*.* Cronbach’s alpha = 0.89.

##### Anxiety and depressive symptoms

The PROMIS Anxiety Short-form [42] has eight items assessing anxiety (e.g., “In the past 7 days, I felt uneasy) on a 5-point Likert scale. Cronbach’s alpha = 0.95. The PROMIS Depression Short-form [42] has eight items assessing symptoms of depression (e.g., “In the past 7 days, I felt depressed) on a 5-point Likert scale. Cronbach’s alpha = 0.93. A *t*-score was calculated for both scales for analyses, and *t*-scores 55–60 indicate mild symptoms, 60–70 indicate moderate symptoms, and greater than 70 indicate severe symptoms.

#### Outcome measures

##### Activation

Activation was defined as understanding one’s role in the care process and having the knowledge, skill, and confidence to take on a role in managing lymphoma self-care. We adapted the Patient Activation Measure (PAM) [2, 43] for this study to focus on the management of lymphoma. The scale contained 12 items (sample items: “When all is said and done, I am the person who is responsible for managing my lymphoma care,” “I am confident that I can take actions that will help prevent or minimize some symptoms or problems associated with my lymphoma care”). The response scale was modified to a 4-point Likert scale. Cronbach’s alpha was 0.88. For purposes of analyses, the mean score was transformed into a standardized activation score ranging from 0 to 100 based on a conversion table provided by the developer [2, 43]. The PAM score was then converted into one of the four levels of patient activation (1 = not yet taking an active role in care, 2 = gaining confidence and knowledge to take action, 3 = taking action, and 4 = maintaining behaviors) [44].

##### Preparedness for survivorship

Preparedness was defined as the extent to which individuals perceived they have sufficient information about what to expect after cancer treatments are completed. Eight items adapted from Manne and colleagues [45] assessed whether information received about survivorship care was sufficient, easy to understand, helpful, addressed needs, and addressed how to manage symptoms, look for signs of cancer, and self-care. These items were rated on a 5-point Likert scale*. An average score was used in the analyses.* Cronbach’s alpha = 0.94.

### Data analytic approach

Descriptive information (frequencies/percentages, means, standard deviations) was used to characterize levels of activation and preparedness. Given the large number of possible predictors, we adopted a two-step approach for the analyses. First, we conducted separate regression models predicting preparedness and activation in which all variables within a particular domain (e.g., all sociodemographic or all medical variables) were treated as predictors. We accounted for patient clustering within physicians using Generalized Estimating Equations with robust standard errors [46]. Using the results from these analyses, we selected only those variables that attained *p* < 0.05 statistical significance to include in the primary analysis. The primary analysis was a multi-domain hierarchical multiple regression arranged in the following four steps: sociodemographic variables, medical, survivorship care experiences, and psychosocial characteristics. Change in marginal *R*^2^ [47] was computed for each step. We assessed model assumptions using standard linear regression diagnostics. We assessed robustness to deviations from model assumptions by using the Box-Cox transformation for preparedness and activation.

## Results

### *Sociodemographic and medical characteristics (*Table 1*)*

**Table 1:** Sociodemographic and medical characteristics of the sample

Domain	*N* (%)	*M* (SD)
**Sociodemographic domain**		
**Sex**		
Male	52 (51.5)	
Female	49 (48.5)	
**Age (years)**		50.6 (16.1)
**Race**		
White	83 (82.2)	
Black	5 (4.9)	
Asian or Asian American	8 (7.9)	
American Indian/Alaska native/Native Hawaiian	0 (0.0)	
Other	5 (4.9)	
**Hispanic ethnicity**		
Yes	18 (17.8)	
No	81 (81.2)	
Do not know	1 (1.0)	
**US-born**		
Yes	84 (83.2)	
No	17 (16.8)	
**Marital status**		
Married	63 (62.4)	
Widowed	4 (4.0)	
Divorced/separated	13 (12.9)	
Single	19 (18.8)	
Prefer not to answer	2 (2.0)	
**Education**		
High school graduate or less	9 (22.6)	
Some college/post-high school	30 (24.0)	
College graduate	38 (30.3)	
Graduate degree	24 (21.4)	
**Employment status**		
Employed/homemaker/student/part-time	68 (45.4)	
Unemployed/retired/disabled	28 (52.0)	
Prefer not to answer	2 (2.7)	
**Household income**		
< $10,000	2 (2.0)	
$10,000–$19,999	2 (2.0)	
$20,000–$29,999	8 (7.9)	
$30,000–$39,999	2 (2.0)	
$40,000–$49,999	6 (5.9)	
$50,000–$59,999	5 (5.4)	
$60,000–$69,999	7 (6.4)	
$70,000–$79,999	8 (5.0)	
$80,000–$89,999	3 (5.3)	
> $90,000	45 (44.6)	
Prefer not to answer/missing	13 (10.9)	
**Health insurance status**		
Private	60 (59.4)	
Medicaid	14 (13.9)	
Medicare	22 (21.8)	
Other	4 (4.0)	
None	1 (1.0)	
**Area Deprivation Index**		
1 (most deprived)	12 (11.9)	
2	19 (18.8)	
3	13 (12.9)	
4	12 (11.9)	
5	10 (9.9)	
6	11 (10.9)	
7	9 (8.9)	
8	6 (5.9)	
9	4 (4.0)	
10 (least deprived)	5 (5.0)	
**Medical domain**		
**Primary cancer diagnosis**		
Hodgkin’s lymphoma	36 (35.6)	
Non-Hodgkin’s lymphoma	63 (62.4)	
**Surgery**		
Yes	1 (1.0)	
No	100 (99.0)	
**Chemotherapy**		
Yes	86 (14.9)	
No	15 (85.1)	
**Radiation**		
Yes	3 (3.0)	
No	98 (97.0)	
**Time since diagnosis (months)**		3.34 (2.66)
**Comorbidities**		
0	46 (45.5)	
1	19 (18.8)	
2	17 (16.8)	
3	14 (13.9)	
4	2 (2.0)	
> 5	3 (1.0)	
**Fatigue**		3.65 (2.31)
**Sleep difficulties**		11.4 (3.65)

One hundred and one survivors participated, ranging in age from 21 to 78 years (*M* = 51 years). The majority were White (82%), not Hispanic (82%), and born in the US (83%). Approximately half completed at least a college-level education, and all but one participant carried health insurance.

### *Care transition, practical, and psychosocial characteristics (*Table 2*)*

**Table 2:** Care transition experiences, practical, psychosocial domain, and outcomes

Domain	*N* (%)	*M* (SD)
**Care transition experiences**		
Discuss reasons for follow-up care		3.19 (2.35)
Discuss late effects and other health topics		1.84 (0.60)
Care transition practices		2.66 (1.04)
**Practical domain**		
**Financial hardship**		1.09 (1.22)
0	43 (42.6)	
1	28 (27.7)	
2	14(13.9)	
3	10 (9.9)	
4	6 (5.9)	
**Information needs**		4.65 (3.46)
**Psychosocial domain**		
Support needs		5.00 (5.81)
Fear of recurrence		3.39 (1.52)
Cancer concerns		2.22 (0.87)
Depressive symptoms		48.05 (8.93)
Anxiety		53.15 (10.23)
**Outcomes**		
Preparedness		2.94 (0.79)
Activation		3.08 (0.46)
1 (Lowest activation)	17 (16.8)	
2	25 (24.8)	
3	28 (27.7)	
4 (Most activation)	30 (29.7)	

Discussions regarding reasons for follow-up care were common, with a median of three reasons provided. Care transition practices were not as common: Only 38.6% reported they had been provided with access to a navigator, and about half (51.5%) reported receiving a treatment summary. Information needs nominated most frequently were as follows: “Late and long-term side effects to expect” (61.4%), “Cancer-related follow-up tests I should have” (59.4%), and “Medical advances in cancer treatment” (55.4%). The most common unmet support needs were psychosocial: uncertainty about the future (28.7%), fear of the cancer spreading (27.7%), anxiety (23.8%), help with assistance with handling worries of family and friends (23.8%), worry that the results of the cancer are beyond control (21.8%), depression (19.8%), learning to feel in control of the situation (18.8%), and sadness (17.8%). Average levels of fear about recurrence were midrange (*M* = 3.4; range, 1–6). The average level of concern was 2.2 (2 = *a little concerned*). Depression was primarily in the normal range (76.8%), with 13.9% reporting mild, 8% reporting moderate, and 1% reporting severe symptoms. Anxiety was higher, with 56.4% in the normal range, 14.9% reporting mild, 22.7% reporting moderate, and 6.0% reporting severe anxiety.

### Characterizing activation and preparedness

Table 2 shows the means for the activation and preparedness. Levels of activation were distributed relatively evenly, with about 41.6% reporting not taking an active role in their care or gaining confidence and knowledge to take action and about 57.4% reporting taking actions or maintaining behaviors [44]. Among the items, the lowest activation means were reported for: “I know how to prevent further problems” (51.1% *disagree/strongly disagree*), “I know the recommended self-care practices” (35.8% *disagree/strongly disagree)*, and “I can figure out solutions when new situations or problems arise” (33% *disagree/strongly disagree*). The remaining scores were all between 5 and 19% *disagree/strongly disagree*.

Average preparedness was relatively high (*M* = 2.94 on a 4-point scale, 3 = s*omewhat agree*). The lowest levels of preparedness were reported for: “The information I received has covered helpful daily exercises to increase or maintain physical function” (45.9% *somewhat/strongly disagree*) and “The information I received has covered how to look for signs of cancer” (42.4% *somewhat/strongly disagree*). The highest preparedness was “The information has outlined the follow-up care schedule” (15.8% *somewhat/strongly disagree*).

### Multiple regression predicting activation

The initial models for activation are shown in the top panel of Table 3. Variables associated with significantly higher activation were older age, more scores on care transition practices, higher scores on provider discussion of health topics, and greater fear of recurrence. Variables associated with lower activation were being currently married, living in a more deprived geographic area, higher fatigue, and higher informational needs.

**Table 3:** Initial regression models predicting activation and preparedness

	Estimate	SE	95% CI		Pr ( >|W|)
Dependent variable: activation					
Sociodemographic domain					
(Intercept)	54.9484	5.5392	44.092	65.805	0
Age	0.2467	0.0789	0.092	0.401	0.0018
Gender = 2 (ref = 1/male)	− 0.6424	1.3022	− 3.195	1.910	0.6218
Ethnicity = 1 (ref = 0)	5.3543	5.9394	− 6.287	16.996	0.3673
White race (vs all other)	− 3.1714	2.7757	− 8.612	2.269	0.2532
Marital status (yes vs no)	− 5.2867	2.0516	− 9.308	− 1.266	0.01
Employment status (yes vs no)	1.1207	1.8379	− 2.482	4.723	0.542
Area Deprivation Index	− 1.1721	0.1567	− 1.479	− 0.865	0
Medical domain					
(Intercept)	75.685	2.609	70.571	80.799	0
Had surgery	− 1.924	1.929	− 5.705	1.857	0.319
Had radiation	− 4.671	3.065	− 10.678	1.336	0.128
Had chemotherapy	− 4.808	2.751	− 10.200	0.584	0.08
Comorbidity	− 0.254	0.723	− 1.671	1.163	0.725
Fatigue	− 1.763	0.485	− 2.714	− 0.812	0
Sleep difficulty	− 0.233	0.179	− 0.584	0.118	0.192
Care transition experiences domain					
(Intercept)	40.486	4.199	32.256	48.716	0
Discuss reasons for follow-up care	− 0.455	0.726	− 1.878	0.968	0.53
Discuss late effects and other health topics	8.387	2.696	3.103	13.671	0.002
Care transition practices	2.201	0.827	0.580	3.822	0.008
Practical domain					
(Intercept)	64.471	1.906	60.735	68.207	0
Financial hardship	− 0.614	0.532	− 1.657	0.429	0.249
Information needs	− 0.788	0.354	− 1.482	− 0.094	0.026
Psychosocial domain					
(Intercept)	90.456	11.043	68.812	112.100	0
Support needs	− 0.501	0.307	− 1.103	0.101	0.103
Fear of recurrence	0.875	0.496	− 0.097	1.847	0.078
Cancer concerns	− 0.987	3.954	− 8.737	6.763	0.803
Anxiety	− 0.178	0.102	− 0.378	0.022	0.081
Depressive symptoms	− 0.391	0.333	− 1.044	0.262	0.242
Dependent variable: preparedness					
Sociodemographic domain					
(Intercept)	2.854	0.291	2.284	3.424	0
Age	0.009	0.008	− 0.007	0.025	0.258
(Intercept)	− 0.133	0.12	− 0.368	0.102	0.268
Age	− 0.318	0.166	− 0.643	0.007	0.055
Gender = 2 (ref = 1)	0.012	0.123	− 0.229	0.253	0.924
Ethnicity = 1 (ref = 0)	0.21	0.101	0.012	0.408	0.037
White race (vs all other)	− 0.317	0.114	− 0.540	− 0.094	0.005
Area Deprivation Index	0.029	0.02	− 0.010	0.068	0.143
Medical domain					
(Intercept)	3.479	0.145	3.195	3.763	0
Had surgery	− 0.177	0.058	− 0.291	− 0.063	0.002
Had radiation	− 0.021	0.156	− 0.327	0.285	0.891
Had chemotherapy	0.079	0.148	− 0.211	0.369	0.592
Comorbidity	0.099	0.029	0.042	0.156	0.001
Fatigue	− 0.03	0.025	− 0.079	0.019	0.218
Sleep difficulty	− 0.05	0.024	− 0.097	− 0.003	0.035
Care transition experience domain					
(Intercept)	1.342	0.126	1.095	1.589	0
Discuss reasons for follow-up	0.045	0.016	0.014	0.076	0.005
Discuss late effects and other health topics	0.582	0.067	0.451	0.713	0
Care transition practices	0.138	0.025	0.089	0.187	0
Practical domain					
(Intercept)	3.128	0.129	2.875	3.381	0
Financial hardship	− 0.037	0.043	− 0.121	0.047	0.389
Information Needs	− 0.032	0.011	− 0.054	− 0.010	0.004
Psychosocial domain					
(Intercept)	3.589	0.296	3.009	4.169	0
Support needs	− 0.067	0.011	− 0.089	− 0.045	0
Fear of recurrence	0.012	0.047	− 0.080	0.104	0.794
Cancer concerns	0.2	0.139	− 0.072	0.472	0.15
Anxiety	− 0.017	0.01	− 0.037	0.003	0.073
Depressive symptoms	0.002	0.015	− 0.027	0.031	0.869

The final model is shown in the top panel of Table 4. Across all domains, age, marital status, area-level deprivation, fatigue, provider discussion of health topics, care transition practices, and informational needs remained significant in the final model. The inclusion of the medical domain and the care transition domains was associated with the largest increases in marginal *R*^2^ (0.11 and 0.13, respectively). Results were largely robust after applying the Box-Cox transformation to the activation outcome, although information needs were not significantly associated with transformed activation.

**Table 4:** Final multivariate models predicting activation and preparedness

Domain	Predictors	Estimate	SE	95% CI		*p*-value	Marginal R2
**Dependent variable: activation**							
	(Intercept)	49.37	7.85	33.984	64.756	< 0.001**	
**Demographic**	Age	0.206	0.043	0.122	0.290	< 0.001**	0.056
	Marital status	− 6.899	2.118	− 11.050	− 2.748	0.001**	
	Area deprivation	− 1.201	0.105	− 1.407	− 0.995	< 0.001**	
**Medical**	Fatigue	− 2.121	0.232	− 2.576	− 1.666	< 0.001**	0.166
**Care transition experiences**	Discuss late effects and other health topics	8.758	4.036	0.847	16.669	0.03**	0.292
	Care transition practices	1.695	0.619	0.482	2.908	0.006**	
**Practical**	Information needs	− 0.602	0.3	− 1.190	− 0.014	0.045*	0.307
**Psychosocial**	N/A						
**Dependent variable: preparedness**							
	(Intercept)	2.504	0.162	2.186	2.822	< 0.001**	
**Demographic**	Employment status	− 0.283	0.08	− 0.440	− 0.126	< 0.001**	0.052
**Medical**	Comorbidities	0.049	0.028	− 0.006	0.104	0.077	0.131
	Had Surgery	− 0.118	0.066	− 0.247	0.011	0.077	
	Fatigue	− 0.045	0.014			0.002**	
**Survivorship care experiences**	Discuss reasons for follow-up	0.061	0.027	0.008	0.114	0.024**	0.464
	Discuss late effects and other health topics	0.48	0.132	0.221	0.739	< 0.001**	
	Care transition practices	0.078	0.029	0.021	0.135	0.006**	
**Practical**	Information needs	0	0.007	− 0.014	0.014	0.978	0.471
**Psychosocial**	Support needs	− 0.038	0.01	− 0.058	− 0.018	< 0.001**	0.519

Note. *For Activation, after variable transformation, information needs was no longer significant (*p* = 0.053)

Note. **Significant value

### Multiple regression predicting preparedness

The initial models for preparedness are shown in the bottom panel of Table 3. Variables associated with higher preparedness were more comorbidities, sleep problems, more discussion about reasons for follow-up care, more care transition practices, and more discussion about other health topics. Variables associated with significantly lower preparedness were being employed, receiving surgical treatment, higher informational needs, and higher support needs.

The final model is shown in the bottom panel of Table 4. Employment, sleep problems, all three care transition practices, and support needs remained statistically significant in the final model. The care transition practice domain led to a large increase in marginal *R*^2^ (0.33, or 33% of variance), and the medical domain also resulted in a notable increase in marginal *R*^2^ (0.08, or 8% of variance). Although preparedness was found to be left-skewed, these results were robust after applying a normalizing Box-Cox transformation; the direction was unchanged, and no variables lost significance.

## Discussion

In this paper, we characterized and examined correlates of patient activation and preparedness among lymphoma survivors who completed treatment within the last 5 years. Results indicated that almost 60% of survivors reported high levels of activation, with similar percentages for higher levels of preparedness. Survivors reported the lowest activation for preventing future cancer-related problems and managing self-care and the lowest preparedness for recognizing signs of cancer recurrence and methods to increase or maintain physical function. Less activated survivors were younger, married, resided in a more deprived geographic area, reported more fatigue, and more information needs. Their providers were less likely to discuss late or long-term side effects, psychosocial needs, risk-reducing lifestyle or health recommendations, and how to manage other medical concerns. Finally, fatigue was a significant correlate of low activation. The levels of activation in this study were lower than those reported in prior research. In a study focusing on breast and prostate cancer survivors recruited from a community oncology setting, who were between 2 and 10 years post-treatment, O’Malley and colleagues [18] reported that 14% of their sample was in the lowest two activation levels. In Aquati and colleagues’ study of mixed solid tumor survivors, 8.5% of the sample was in the lowest two activation levels. In contrast, we reported that 42% were in the lowest two levels. One potential explanation for these differences is that our population was composed of patients who were diagnosed earlier in the survivorship trajectory. More than half of O’Malley and colleagues’ sample [18] completed treatment between 2 and 5 years, with the remaining completing treatment more than 10 years prior to participation. In our sample, 31% were diagnosed within the last 2 years and almost 80% were diagnosed within the last 3 years.

Because we assessed the time since *diagnosis* rather than the time since treatment *completion*, our figure would be lower once the treatment phase was included in the calculation. However, this explanation may not explain the current finding as the time since diagnosis reported by Acquati and colleagues [48] was more similar to our figures, with about 27% diagnosed within the last 2 years. Low activation may be, in part, due to typical oncology care provider practices for this patient population. For most patients, providers consider 3 or more years without a recurrence or progression as “survivorship.” This is supported by our data suggesting that almost half of the sample did not receive a survivorship care plan or treatment summary or did not know if they did, 60% desired more information about cancer-related follow-up tests they should have, and 63% desired more information about long-term side effects. It is possible that providers were not transitioning their patients into survivorship after treatment was completed.

Our findings about associated factors are consistent with O’Malley and colleagues [18], who also found that married prostate cancer survivors reported lower activation, but not consistent with Aquati and colleagues [48], who reported that female survivors were more activated. Our findings extend prior work in other cancer survivor populations by finding that less activation was seen in younger lymphoma survivors, survivors living in more deprived geographic areas, and survivors reporting more fatigue and higher information needs. Most importantly, our findings point to the important role of oncology care providers in activating their patients by providing information and recommendations for follow-up care and the importance of engagement in healthy lifestyle behaviors.

Greater preparedness was primarily associated with care transition practices, including more patient-recalled discussions about reasons for follow-up care, engagement in more care transition practices, and discussions about what to expect in terms of treatment effects and maintaining good health and well-being. Finally, lower preparedness was associated with being employed and higher support needs. Our finding regarding the importance of care transition practices is consistent with prior work [10, 20]. However, unlike Leach and colleagues [10] and Fong and colleagues [20], we did not find an association between area-level deprivation and preparedness. Further, anxiety, depression, and fear of cancer recurrence were not associated with either outcome in the final regression analyses.

This study has limitations. We employed a cross-sectional study design, and therefore, we cannot make causal inferences. Participants provided retrospective ratings of providers’ care practices that occurred up to several years prior to survey completion, which may be biased by post-treatment experiences, and participants may not have been able to recall all conversations they had with their providers accurately. A prospective, longitudinal study characterizing survivorship care experiences over time may better illuminate the association between providers’ care practices, post-treatment experiences, and activation and preparedness. Additionally, with our relatively limited sample size, we were not able to control for multiple testing, although we did follow a pre-specified analytic plan to increase rigor. Future studies with larger samples should be conducted to confirm associations. At approximately 32%, the study acceptance rate was not high. The sample was small and drawn from a single cancer center, which may limit generalizability. In terms of representativeness of our sample, although there was a distribution of survivors residing in more deprived geographic areas and more than half reported financial hardships, the majority were born in the US and almost all were insured. It is possible that participants had higher socioeconomic status than refusers, but we did not collect this information on study refusers. Compared with both state and national figures, our sample was younger than state and national figures and comprised more Hispanics compared with state statistics [49, 50]. Patients with non-Hodgkins lymphoma were much more likely to refuse participation than Hodgkin’s lymphoma survivors. Although we can only speculate, post-hoc comparisons of HL and NHL groups in our sample indicated that HL survivors were significantly younger, more anxious, and more depressed than NHL survivors. Mental health concerns may have contributed to the decision to decline participation in this study. Although there has been a broad range of incentives for cross-sectional studies reported in the literature, with some studies reporting incentives similar or greater than our incentive of $40 [51–53], our study incentive may be high for a cross-sectional study. In future work, qualitative data from interviews with patients and providers may elucidate preferences for how survivorship care planning and post-treatment care processes can be enhanced for lymphoma survivors.

## Implications for cancer survivors

Our findings have potential implications for how oncology care providers and survivorship programs can foster a higher-quality transition for patients coming off active treatment in early survivorship. First, because patient-recalled care transition practices were responsible for a relatively substantial increase in variance, both oncology care and non-oncology care providers should be educated about the importance of these practices. Second, at the end of active treatment and routinely over the survivorship trajectory, oncology care and non-oncology providers should uniformly discuss reasons for follow-up, discuss follow-up appointment schedules, provide a treatment summary, and offer survivorship navigation services. Third, oncology providers engaged with recent lymphoma survivors should routinely ask about their patients’ confidence in managing their care and their preparedness and provide referrals for appropriate care as needed. Fourth, lymphoma survivors who are younger, employed, married, living in socio-demographically deprived areas, experiencing fatigue, and reporting more information and support needs should be specifically targeted for a more intensive approach to transitioning to survivorship. A number of survivorship transition models have been developed that focus on providing education about treatment effects [13]. The finding that area-level deprivation is associated with outcomes suggests a more comprehensive assessment of geographic factors may be important, particularly since these survivors may need community-focused approaches to improve their outcomes. Finally, survivor-focused intervention studies are needed to determine the most effective solutions to improving lymphoma survivorship care.

## Data Availability

The datasets generated during and/or analysed during the current study are available from the corresponding author on reasonable request.
